# Soil Microbiome Is More Heterogeneous in Organic Than in Conventional Farming System

**DOI:** 10.3389/fmicb.2016.02064

**Published:** 2017-01-04

**Authors:** Manoeli Lupatini, Gerard W. Korthals, Mattias de Hollander, Thierry K. S. Janssens, Eiko E. Kuramae

**Affiliations:** ^1^Department of Microbial Ecology, Netherlands Institute of Ecology (NIOO-KNAW)Wageningen, Netherlands; ^2^Department of Terrestrial Ecology, Netherlands Institute of Ecology (NIOO-KNAW)Wageningen, Netherlands; ^3^MicroLife SolutionsAmsterdam, Netherlands; ^4^Department of Ecological Science, Vrije Universiteit AmsterdamAmsterdam, Netherlands

**Keywords:** soil health treatment, soil-borne pathogen, sustainability, agro-ecosystem, 16S rRNA, bioindicator, microbial ecology, microbial diversity

## Abstract

Organic farming system and sustainable management of soil pathogens aim at reducing the use of agricultural chemicals in order to improve ecosystem health. Despite the essential role of microbial communities in agro-ecosystems, we still have limited understanding of the complex response of microbial diversity and composition to organic and conventional farming systems and to alternative methods for controlling plant pathogens. In this study we assessed the microbial community structure, diversity and richness using 16S rRNA gene next generation sequences and report that conventional and organic farming systems had major influence on soil microbial diversity and community composition while the effects of the soil health treatments (sustainable alternatives for chemical control) in both farming systems were of smaller magnitude. Organically managed system increased taxonomic and phylogenetic richness, diversity and heterogeneity of the soil microbiota when compared with conventional farming system. The composition of microbial communities, but not the diversity nor heterogeneity, were altered by soil health treatments. Soil health treatments exhibited an overrepresentation of specific microbial taxa which are known to be involved in soil suppressiveness to pathogens (plant-parasitic nematodes and soil-borne fungi). Our results provide a comprehensive survey on the response of microbial communities to different agricultural systems and to soil treatments for controlling plant pathogens and give novel insights to improve the sustainability of agro-ecosystems by means of beneficial microorganisms.

## Introduction

Over the past decades, anthropogenic alteration of soils by the increased use of synthetic fertilizers, pesticides and land conversion in order to increase food production is causing unprecedented changes in biodiversity, and thus, rising concern on the sustainability of intensive farming systems. The agriculture intensification has a substantial impact on plant and animal diversity (Gabriel et al., 2006; Jonason et al., 2011). However, the effects of agricultural management on below-ground diversity are not well understood (Li et al., 2012). This lack of knowledge is a significant concern because soil-borne microbes, especially bacteria, represent the majority of biodiversity in soil ecosystems and are involved in multiple ecosystem functions, including nutrient cycling (Pan et al., 2014; Navarrete et al., 2015) and plant health (Mazzola, 2004; Wakelin et al., 2013).

The environmental problems associated with the intensification of agriculture have initiated research efforts for conservation strategies. Converting conventional farms to organic farming systems seems to be a potential solution to diminish the loss of biodiversity and increase sustainable food production (Gonthier et al., 2014). Organic farming system consists of low-input agro-ecosystem farms in which plant productivity and ecosystem functionality are based on the natural availability of plant nutrients, use of green manure and biological pathogen control (Lammerts van Bueren et al., 2002). In contrast, conventional farming system relies on intensive use of agrochemicals, such as synthetic fertilizers to increase crop productivity and use of fungicides and pesticides to promote plant protection against pathogens (Kremen and Miles, 2012). Effects of farming systems on microbial communities are complex and time-dependent (Jonason et al., 2011). In general, it has been reported that management practices in organic farming systems change the microbial composition toward a more fast growing community (copiotrophic community) due to nutrients (Chaudhry et al., 2012), promote habitat diversification, increase the diversity and sustainability, and benefit microbial taxa involved in plant health when compared to conventional farming systems (Esperschutz et al., 2007; Sugiyama et al., 2010; Reilly et al., 2013; Gonthier et al., 2014). However, up to date, there are no studies about microbial community heterogeneity, which we refer as microbial community variability, in different farming systems. Although positive effects of organic management have been widely reported (Liu et al., 2007; Ge et al., 2008; Jonason et al., 2011; Hartmann et al., 2015), the effects of farming systems on diversity of microbial communities are complex and commonly controversial (Kleijn et al., 2001). Ge et al. (2008) found an increase in diversity after manure amendment, and other studies reported no differences or decrease in bacterial diversity and richness when organic systems were compared to conventional management (Liu et al., 2007; Reilly et al., 2013). Bengtsson et al. (2005) argue that in most cases, organic farming can be expected to benefit the biodiversity, but the effects will differ between organism groups and landscapes.

Agro-ecosystems often face problems with plant-pathogens, such as parasitic nematodes (e.g., Pratylenchidae and Meloidogynidae), and soil-borne fungi (e.g., *Rhizoctonia solani* and *Verticillium dahliae*) that affect a large number of important crops (Back et al., 2002). A common method to control these pathogens is the use of chemical pesticides, which are under critical review due potential toxic effect on non-target organisms and environmental pollution (Oka, 2010). Therefore, the development of methods for suppression of pathogens as an alternative to chemical control is an urgent need. These methods can be applied in organic farming systems, but also enable conventional farmers to reduce the use of pesticides. Alternative approaches are organic amendments (compost) (Mehta et al., 2014), cover crops (Asteraceae plants) (Pudasaini et al., 2006), green manure crops (grass-clover) (Widmer and Abawi, 2002), composts or non-composted waste products (chitin) or those based on physical methods (soil disinfestations) (Mowlick et al., 2012). Although these management practices are environmentally friendly, they are expected to induce shifts on microbial diversity and composition (Mehta et al., 2014). At the treatment level, the microbes play an important role in above- and below- ground processes, including their potential contribution to soil suppressiveness (Cretoiu et al., 2013). In this light, the ability to understand and manage microbial community through alternative practices for pathogen control, offer a promising approach to improve sustainable crop production.

The broad spectrum of agricultural managements and practices used for plant pathogen control in farming systems limits comparability among different studies (Liu et al., 2007; Xue et al., 2013). Up to date, there are few long-term agro-ecosystems experiments comparing organic and conventional farming systems (Esperschutz et al., 2007), and even more seldom are studies that make this comparison on plant pathogen control. This would be ultimately required for evaluating the sustainability of agricultural practice. One exception is the experimental field with Soil Health Treatments (SHTs) in organic and conventional farming initiated in 2006. The SHT experimental site in Vredepeel is a unique experimental field reported in contemporary literature with full-factorial experimental design and replicated experimental plots, where the same soil treatments, crop varieties, crop rotations and fertilization intensities are simultaneously applied in both conventional and organic farming systems under the same sandy soil type. Korthals et al. (2014) have evaluated the potential effects of the different SHTs on plant-parasitic nematode *Pratylenchus penetrans*, and on soil-borne pathogenic fungus *V. dahliae*. However, the long lasting responses of the soil microbial community to those different managements and the potential role of microbial community in soil suppressiveness were not studied. In this context, we assessed the bacterial and archaeal communities based on 16S rRNA gene marker by next generation sequencing to examine the response of microbial communities to conventional and organic farming systems and SHTs. The objectives of this study were to address the effect of farming systems and SHTs on (i) soil microbial diversity and presence of pathogen suppressors, and (ii) microbial community heterogeneity. Based on microbial community assessment, we aimed to detect specific structural shifts and identify microbial taxa associated with specific farming system or SHT, which might be useful as a bioindicator of sustainable management of agro-ecosystems and might bring novel insights on soil beneficial agriculture practices for soil health and plant productivity.

## Materials and Methods

### The Soil Health Experiment, Experimental Design and Historical Management

The Soil Health Experiment (SHE) is located at Wageningen University Research (WUR) station in Vredepeel, in the south–east of the Netherlands (51° 32′ 27.10″ N and 5° 51′14.86″ E). The site has been in agricultural cultivation since 1955, and has a mean annual air temperature of 10.2°C and mean annual precipitation of 766 mm. This experiment is unique since all crops and treatments applied are compared simultaneously on the same soil type (sandy soil: 1.1% clay, 3.7% silt and 94.9% fine sand) and in which conventional and organic farming systems differ only in fertilization and plant protection methods. In spring 2006, the experimental field was divided into 160 plots, each 6 m × 6 m and arranged in a randomized block design with four replicates. Within each block, two agricultural farming systems, conventional and organic, were randomized. Each year between 2006 and 2013, a crop was grown on the entire experimental field: 2006: Wheat (Conv) or barley (Org); 2007: potato (Conv, Org); 2008: lily (Conv, Org); 2009: Wheat (Conv) or barley (Org); 2010: potato (Conv, Org), 2011: carrot (Conv, Org), 2012: maize (Conv, Org), 2013: maize (Conv, Org). Both systems received the same amount of Nitrogen, Potassium, and Phosphorus nutrients per hectare and year according to fertilizer recommendations for the crops. The organic system exclusively received organic fertilizers, whereas conventional system was based in a fertilization scheme combining organic and mineral fertilizers. In April 2013, initial fertilization was carried out with cattle slurry. One month later, mineral fertilizers were applied in the conventional system, and farm yard manure was applied in 17th of April in the organic system (details on nutrients inputs for the conventional system are in Korthals et al., 2014). In conventional system the plant protection was performed using herbicides, fungicides and insecticides according to the thresholds for each crop (following the rules of European Union). In the organic system, the mechanical weeding was performed. For a complete description of the experimental field and the main conclusion of the previous study, see Korthals et al. (2014).

The SHTs are applied in every 3–4 years, depending on the specific crop-rotation scheme. This frequency is to reach the best cost-effective treatment that can be applied by farmers. Since the beginning of the experiment (2006), the Soil Health Treatments (SHTs) were applied two times until 2013, the year in which the soil sampling for this study was performed. From the end of July 2006 till May 2007, nine different SHTs were applied (**Table 1**). The SHTs were applied for the second time from the end of July 2009 till December 2009 as described for 2006.

**Table 1:** Soil heat treatments applied in conventional and organic systems.

Treatment	Quantity	Material	Soil incorporation (cm)
Compost (CO)	50 Kg/ha	Compost (65% wood, 10% leaves and 25% grass, inoculated with *Trichoderma harzianum*)	20
Chitin (CH)	20 Kg/ha	Chitin-rich shrimp debris (Gembri)	20
Marigold (MA)		*Tagetes patula* (cv. Ground Control)^a^	20
Grass-Clover (GC)	22 Kg/ha	Mix of 4 rye grass and 2 clover species^b^	20
Biofumigation (BF)	117 Kg/ha	Broccoli (cv. Montop)^c^	20
Soil anaerobic disinfestation (AD)	50 Kg/ha	Fresh organic matter, covered plastic^d^	20
Physical control (PH)		Hot air (720–780°C) in humid soil	
Combination (CB)		Combination of MA, CO, CH	20
Chemical control (CC)^∗^	300 L/ha	Methan sodium (Monam 510 g a.i/L)^e^	
Caliente control (CL)^∗^	70 L/ha	Byproduct of mustard product^f^	
Control treatment (CT)		No input	

*^a^*Tagetes patula* biomass from 10 kg/ha seed density grown from July 2006 till January 2007.*

*^b^Rye grass species (4 kg/ha cv. Tetraflorum, 7 kg/ha cv. Miracle, 2 kg/ha cv. Pomposo, 1 kg/ha cv. Tomaso) and clover species (1 kg/ha cv. Riesling, 7 kg/ha cv. Maro) grown from 27 July 2006 till 12 March 2007.*

*^c^Broccoli containing glucosinolates were incorporated and degraded into plant metabolites (i.e., isothiocyanates, nitriles and thiocyanates) with biocidal properties.*

*^d^In August 2006, the incorporated mixture of rye-grass species was irrigated with 20 mm water per plot and covered with a plastic (0.035 mm thick HyTibarrier). In November 2006 the plastic was removed.*

*^e^Application of chemical on September 2006 with a rotary spading injector, a common technique allowed by the Dutch ministry. Only applied in conventional system.*

*^f^Applied only in organic system.*

*^∗^Control treatments (CC, CL). CL treatment was applied only in organic system (no chemical inputs are allowed in organic system) for comparative purpose to CC in conventional system.*

### Soil Sampling, DNA Isolation and 16S rRNA Gene Amplification

Soil sampling was performed in May 2013, therefore represents the soil microbial communities just before another SHT application. Three soil cores (top-layer 0–10 cm) from each SHT plot were sampled and pooled to make a single composite sample, resulting in 60 independent sample plots (2 farming systems × 10 SHT treatments, including controls × 3 replicates). Soil sampling was performed in three blocks and in both conventional and organic systems during the initial stage of maize crop. This sampling scheme was chosen since it reflects the long-term effects of conventional and organic farming systems and the legacy effects of SHTs on microbial communities. Samples were stored at -80°C until DNA isolation process. From each sample 2 g of soil was used for total DNA isolation using the DNA PowerSoil kit (MoBio laboratories, Inc.) and the yield and quality were determined using NanoDrop 1000 spectrophotometer (Thermo scientific, USA). Bacterial and Archaeal communities were determined based on the hypervariable region V4 of 16S rRNA gene using the barcoded primers 515F/806R. A 25 μL reaction was prepared containing 5 μL *Taq* FastStart High Fidelity Enzyme Blend, 10x FastStart High Fidelity Buffer with 1.8 mM MgCl_2_ (Roche Diagnostics Ltd., Burgess Hill, UK), 0.2 mM of each dNTP (Promega UK Ltd. Southampton, UK) with each primer used at 0.1 M. For each reaction 1 μL of DNA template was used. The conditions used were a hot start of 95°C for 5 min, followed by 35 cycles of 95°C for 30 s, 50°C for 30 s and 72°C for 1 min with a final extension at 72°C for 10 min. The PCR reactions were conducted in triplicate. Reactions were amplified in a C1000 Touch thermal cycler (Bio-Rad, Hemel Hempstead, UK). Resultant amplicons were visualized on a 1% (w/v) TBE agarose gel to assess quality of amplicon before pooling the triplicate reactions.

### PCR Purification and Sequencing

The PCR pooled samples were recovered from agarose gel and purified using a QIAquick gel extraction kit (Qiagen). The purified samples were quantified with Quant-iT Broad-Range DNA Assay Kit (Invitrogen) in conjunction with the BioTek Synergy HT microplate reader and combined in equimolar ratios. The 16S rRNA gene fragments were sequenced using Ion Torrent^TM^ semiconductor technology chemistry for unidirectional sequencing of the amplicon libraries. Barcoded primers were used to multiplex the amplicon pools in order to be sequenced together and separated afterward. The barcode of 8 bases was added to the primer 515F and unidirectional sequencing was performed from the A-key adapter. A two-base linker sequence was inserted between the adapter and the 16S rRNA primers to reduce any effect of the composite primer might have on PCR amplication. Template preparation was performed using the Ion OneTouch 2 System and Ion PGM Template OT2 400 Kit, and subsequently sequenced using Ion PGM Sequencing 400 on an Ion PGM System using Ion 318 Chip v2.

### Sequence Data Processing

The 16S rRNA partial gene reads were analyzed using MOTHUR version 1.33.2 (Schloss et al., 2009) combined with the workflow engine Snakemake (Koster and Rahmann, 2012). Briefly, to reduce sequencing errors and their effects, multiplexed reads were first filtered for quality and assigned to samples by matching to barcode sequences. Reads were trimmed including 1 mismatch to the barcode and 2 mismatches to the primer, 8 maximum homopolymer, minimum length of 250 bp, maximun length of 290 and quality score > 25. After trimming, the sequences were aligned using the Silva template (Quast et al., 2013), to eliminate sequences outside of desired range alignment and potentially chimeric sequences were removed using uchime (Edgar et al., 2011). Sequences were classified using Silva rRNA database (release SSU_Ref_119) with a confidence threshold of 80% (35) and the sequences classified as chloroplasts and mitochondria were removed. To build an Operational Taxonomic Unit (OTU) table of each sample and taxonomic assignments for each OTU from 16S rRNA gene, a distance matrix was calculated and sequences obtained were clustered with average neighbor algorithm at a 0.03 dissimilarity threshold. The sequences are available at the European Nucleotide Archive (ENA)^1^ under the study Accession no. PRJEB10907 (ERP012206).

### Statistical Analysis

#### Coverage and Taxonomic Composition

The biom file created on MOTHUR was imported in R (R Development Core Team, 2011) and further analyses using the “phyloseq” package (McMurdie and Holmes, 2013). To estimate how the limited sampling relates to the entirely sampled population, a Good’s coverage estimator (Good, 1953) was calculated at 97% similarity cutoff. Microbial communities at phyla level were compared using a two-way ANOVA after plotting the residuals and confirming the normality of the distribution of the data by Shapiro–Wilk *W*-test (*P* > 0.05) using *shapiro.test* or by Kolmogorov–Smirnov test (*P* < 0.05) using *ks.test*, both tests present in “stats” package. Non-normally distributed data were transformed using the Box-Cox using *boxcox* function in the “MASS” package (Venables and Ripley, 2002) or square root transformed using *sqrt* in the “base” package (R Development Core Team, 2011) in order to achieve the normal distribution of the residuals. When the differences turned out to be significant, they were further analyzed using a post-hoc test by the *HSD.test* (pairwise comparison between treatments, i.e., more than two groups) in the “agricolae” package (de Mendiburu, 2015) and the *pairwise.t.test* (pairwise comparison between systems, i.e., two groups) in the “stats” package. A heatmap was built to visualize the differences in abundance using *heatmap.2* in the “gplots” package (Warnes et al., 2015).

#### Alpha-Diversity

For the estimation of the alpha diversity and richness, the data set was rarefied to 1,691 sequences per sample and three different approaches were employed: (a) community richness was calculated by Observed OTU and ACE estimator, (b) compositional diversity was assessed by applying the Shannon diversity index considering the number and abundance of species using the *estimate_richness* function in the “phyloseq” package; and (c) phylogenetic diversity was calculated by Faith’s phylogenetic diversity index (Faith’s PD) (Faith, 1992) incorporating phylogenetic *distances* between species (*pd* function in the “picante” package (Kembel et al., 2010). The diversity index was analyzed using the two-way analysis of variance (ANOVA) after plotting the residuals and confirming the normality of the data using the Shapiro–Wilk *W*-test. When the differences were significant, they were further analyzed using a *post hoc pairwise.t.test* in the “stats” package.

#### Community Variability (Beta-Diversity)

For further analyses, OTUs with less than 10 sequences were removed. To assess community variability, the absolute number of sequences was transformed to relative abundance and the permutated analysis of betadispersion of pairwise Bray–Curtis (Anderson et al., 2006) and unweight UniFrac similarities using the function *betadisper* in the “vegan” package (Anderson et al., 2006; Oksanen, 2013). The permutation-based hypothesis tests for differences in dispersion of each sample to the group centroid and then tested for differences in these distances between groups. The pairwise comparisons of group mean dispersion were performed by a t-test using *permutest* in the “vegan” package. To visualize significant results, we explored the dissimilarities based on the distance to the centroids determined from the mean positions of the respective samples of conventional and organic systems and plotted in a boxplot.

#### Identification of Strict Habitat Specialists

As higher taxonomic levels provide little information to infer the ecological preferences of the microbial taxa, we decided to identify the *strict habitat specialists* based on OTU level. To test whether a single OTU was associated with either one farming system (conventional or organic) or one soil health treatment, representing habitat types within farming systems, we conducted a species indicator analysis with the *multipatt* function in the “indicspecies” package (De Caceres and Legendre, 2009) in R. This analysis identifies *habitat specialists* based on OTU fidelity (the degree to which an OTU is present at all sites of a defined sample group or habitat) and specificity (the degree to which an OTU is found only in a given sample group or habitat) (Legendre and Legendre, 1998). Because low abundance of individual OTUs is prone to error since it tends to be unique to a habitat and erroneously may indicate a taxa as *strict habitat specialist* (Pandit et al., 2009), we used the same previous data set where OTUs with less than 10 sequences were excluded. Furthermore, a randomized strategy (permutation) was applied to test the probability that an association between an OTU and a habitat (that is, farming system or SHT) was not at random. The statistical significance was tested using 999 permutations. A circular maximum likelihood phylogenetic tree was constructed based on representative sequences for each OTU selected as *strict habitat specialists* between farming system (conventional × organic) and among SHTs within farming system (that is, SHT within Conventional and Organic). The tree was constructed using a distance matrix with relaxed neighbor joining (RNJ) algorithm with the clearcut command (Sheneman et al., 2006) available in MOTHUR and displayed using iTOL (Letunic and Bork, 2007).

## Results

### Number of 16S rRNA Gene Sequences and Coverage

Microbial communities were assessed by sequencing the 16S rRNA gene partial fragment from a short-term experiment with different Soil Health Treatments (SHTs) under conventional and organic farming systems. After quality filtering, a total of 625,298 sequences were obtained from 56 samples with an average of 11,579 sequences (minimum length of 250 bp, maximum length of 290 and quality score > 25) (Supplementary Table S1). Biofumigation treatment was not considered further because less than 300 sequences were recovered per sample (Supplementary Table S1). A total of 3,507 OTUs (with more than 10 reads/sample) were obtained using a 97% identity cut-off. According to Good’s coverage estimator, more than 80% (80–93%) of the OTUs in most of the samples, 77% in one replicate of control organic treatment and 79% in one replicate of caliente organic treatment were captured (Supplementary Table S1).

### Effect of Farming System and SHT on Taxonomic Composition

**FIGURE 1:**
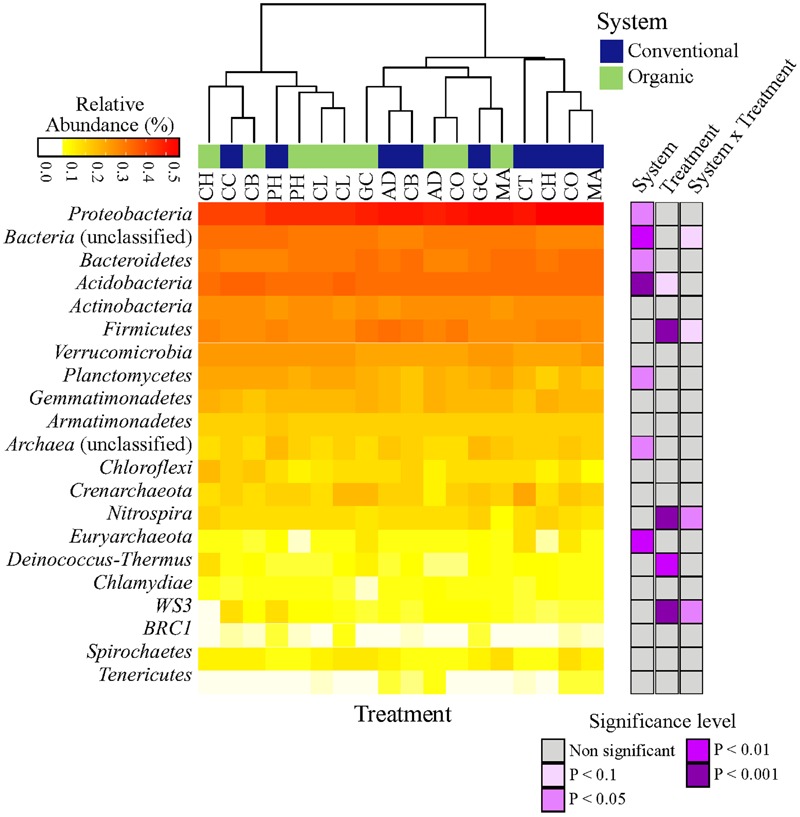
**Heatmap of the response of bacterial community structure at phyla level to farming systems (organic and conventional farming) and Soil Health Treatments (AD, anaerobic soil disinfestation; CC, chemical control; CL, caliente control; CH, chitin; CB, combination; CO, compost; CT, control treatment; GC, grass-clover; MA, Marigold; PH, physical control).** The right panel shows the significance levels (ANOVA test) for systems, SHTs or the interaction between farming systems and SHTs.

The microbial taxonomic composition of different farming systems and SHTs, summarized at phyla level, is shown in **Figure 1**. Overall, a total of 19 phyla (*Archaea* and *Bacteria* domains), 54 classes, 74 orders, 140 families and 230 genera were found within the soil samples. The complete list of all detected bacteria taxa (from Phylum to OTU level) is shown in Supplemental Material 2. Irrespective of systems or treatments, bacterial communities were dominated by *Proteobacteria* (33.80%), *Bacteroidetes* (11.40%), *Acidobacteria* (9.55%), *Actinobacteria* (5.80%), *Firmicutes* (4.30%), *Verrucomicrobia* (2.90%), *Planctomycetes* (2.40%), *Gemmatimonadetes* (1.40%) and *Armatimonadetes* (1.10%). Other phyla were represented by a relative abundance less than 1%. The relative abundances, from highest to lowest abundances of each phylum is shown in **Figure 1**.

The abundances of most bacterial phyla were not statistically different between systems, treatments or the interaction ‘system × treatment’ (**Figure 1**; Supplementary Table S2). Only *Proteobacteria* (ANOVA, *P* < 0.05), *Euryarchaeota* (*P* < 0.01), *Acidobacteria* (*P* < 0.001), and *Planctomycetes* (*P* < 0.05) were significantly affected by farming systems (**Figure 1**; Supplementary Table S2). The relative abundances of *Proteobacteria* (*t*-test, *P* < 0.1) and *Euryarchaeota* (*t*-test, *P* < 0.05) were higher in conventional system, while the abundances of *Acidobacteria* (*t*-test, *P* < 0.05) and *Planctomycetes* (*t*-test, *P* < 0.05) increased in organic system. *Firmicutes, Nitrospira* and *WS3* showed no farming system effect, but *Firmicutes* and *Nitrospira* were more frequent in Anaerobic soil disinfestation and *WS3* was more frequent in physical control, both of them in conventional system (Supplementary Table S2). The effects of farming systems on *Bacteroidetes* (*P* < 0.05) and of treatments on *Deinococcus-Thermus* (*P* < 0.01) were statistically supported by ANOVA, but not by the pairwise comparison. The interaction ‘system × treatment’ on relative abundances of Bacterial unclassified, *Nitrospira* and *WS3* was statistically significant and supported by ANOVA (*P* < 0.1, *P* < 0.05). *Actinobacteria, Verrucomicrobia, Gemmatimonadetes, Armatimonadetes, Crenarchaeota, Chloroflexi, BRC1, Spirochaetes* and *Tenericutes* abundances were not affect by farming systems, treatments nor ‘system × treatment’ interaction (*P* > 0.1).

### Effect of Farming System and SHT on α-Diversity

**FIGURE 2:**
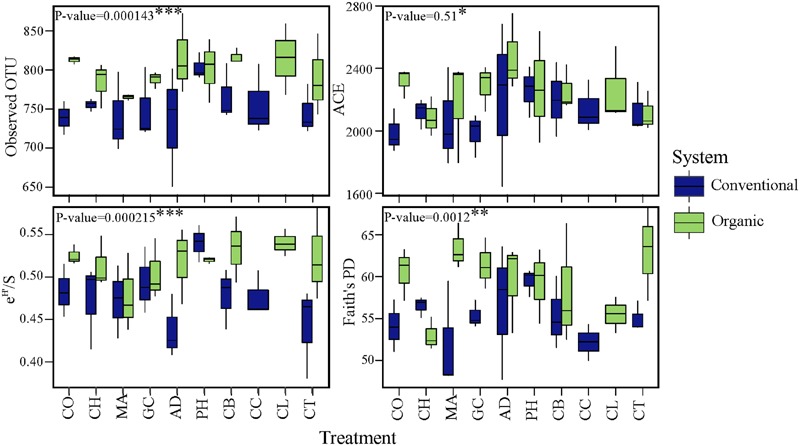
**Effects of farming systems and Soil Health Treatments (AD, anaerobic soil disinfestation; CC, chemical control; CL, caliente control; CH, chitin; CB, combination; CO, compost; CT, control treatment; GC, grass-clover; MA, Marigold; PH, physical control) on bacterial community α-diversities.** On the boxplots, the center lines show the medians, the bottom and upper limits indicate the 25th and 75th percentiles and the whiskers extend 1.5 times the interquartile range from the 25th and 75th percentiles. The values for each diversity index are showed on *y*-axis and STHs on *x*-axis. The significance of the effect of farming systems based on two-way ANOVA on α-diversities.

To investigate changes in microbial diversity in different farming systems and soil treatments, we used taxonomic and phylogenetic metrics approaches. The farming system was a significant driver of microbial taxonomic and phylogenetic α-diversities (ANOVA; Observed OTU and Shannon, *P* < 0.001; Faith’s PD, *P* < 0.05). The α-diversity of microbial community in organic system was significantly higher than in conventional system (**Figure 2**). This result was true for taxonomic observed richness (Observed OTU; 798.5 for organic *vs.* 754 for conventional, *t*-test, *P* < 0.001), taxonomic diversity (Shannon; 6.0 in organic *vs.* 5.8 in conventional, *t*-test, *P* < 0.001) and phylogenetic diversity (Faith’s PD; 59.3 in organic *vs.* 55.2 in conventional, *t*-test, *P* < 0.05). The farming system effect on the α-diversity of bacterial communities based on the ACE estimator was statistically less robust (ANOVA; *P* < 0.1), but a significant pairwise comparison was detected (2250.5 in organic *vs.* 2121.0 in conventional, *t-*test, *P* < 0.05). In contrast to the significant effects of farming system, differences in α-diversity among treatments and the interaction ‘system × treatment’ were small and not significant (*P* > 0.1).

### Farming System and Community Variability

**FIGURE 3:**
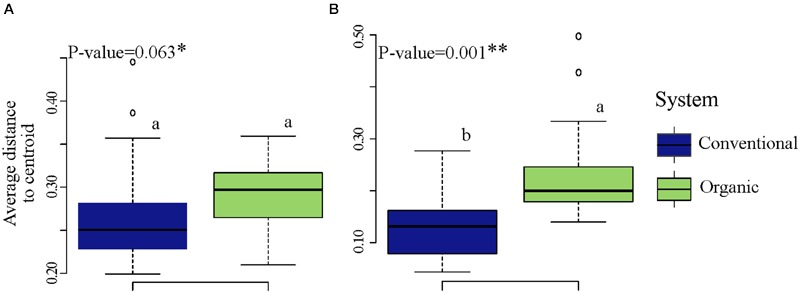
**Variability in bacterial community structure (assessed by analysis of beta dispersion, a metric of variability) in conventional and organic farming systems. (A)** Taxonomic variability; **(B)** Phylogenetic variability. Because the soil health treatments (SHTs) did not show significant effect on community variability (*P* > 0.1), the samples from SHTs were pooled to represent each farming system and the result of beta dispersion was summarized to show only the effects of farming systems. On the boxplots, the center lines show the medians, the bottom and upper limits indicates the 25th and 75th percentiles and the whiskers extend 1.5 times the interquartile range from the 25th and 75th percentiles. Different letters on each box represent significant differences in variance homogeneity between farming systems as determined by HDS-test.

To determine whether microbial community variability (estimated by beta-diversity based on taxonomic and phylogenetic dispersions) were altered by farming systems and/or by SHTs, we used the Bray-Curtis and unweighted UniFrac metric associated with permutest and pairwise comparison. The farming system was a significant driver of microbial taxonomic and phylogenetic variabilities (**Figure 3**), but no significant effects in community dispersion were observed among the treatments within organic and conventional farming systems (*P* > 0.1) (data not shown). The organic farming system had higher effect on community variability than conventional farming, with higher effect on phylogenetic (permutest, *F* = 24.4, *P* < 0.001; **Figure 3B**) than on taxonomic dispersion (permutest; *F* = 3.3, *P* > 0.05; **Figure 3A**).

### Habitat Specialist Taxa of Farming System and SHT

**FIGURE 4:**
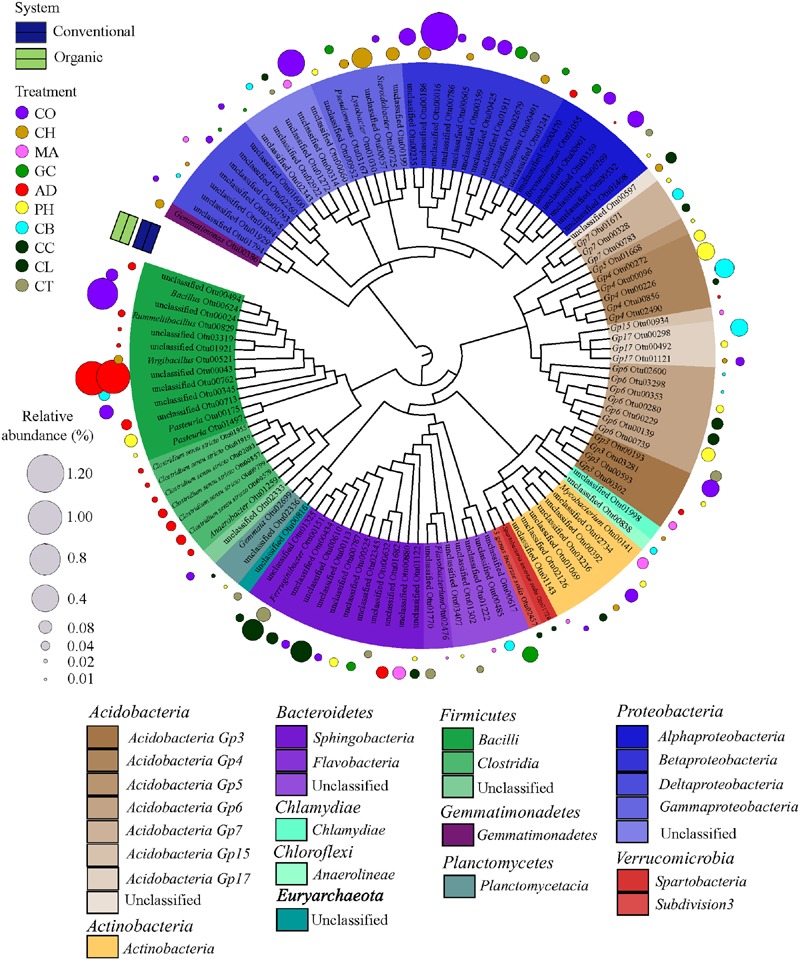
**Dendrogram showing the taxonomy and the habitat specialists associated with soil health treatments (AD, anaerobic soil disinfestation; CC, chemical control; CL, caliente control; CH, chitin; CB, combination; CO, compost; CT, control treatment; GC, grass-clover; MA, Marigold; PH, physical control).** Only the strict specialist OTUs – cut-off 97% – (9.2% of the total OTU data) with statistical significance of the association (*P* < 0.05, *P* < 0.01, and *P* < 0.001) were considered. The taxonomic affiliation at class level of different Phyla of each specialist OTU is identified by the colors range in the below panel and within the tree. The habits preference for a given OTU is indicated in circles outside of the tree. The SHTs within farming systems (conventional is represented by blue and organic by green colors). The diameter of the circles represents the relative abundance (square-root transformed) of the species. Detailed information on abundance of each OTU is provided in Supplementary Material 2.

In order to find the legacy effects of either farming system or SHT, we carried out an indicator species analysis at OTU level, which identifies potential *strict habit specialists* for habitat. The indicator species approach is based on the relative frequency and relative average abundance, identifies a given OTUs that tends to be present mostly in a single habitat type (that is, only in one farming system or SHT) and in most of the samples from that habitat, suggesting the species preference for a given environmental condition. For every OTU identified as specialist, the information on relative abundance of OTUs in each treatment group and taxonomic classification are provided in Supplementary Material 2. Most of the OTUs did not show significant differences in relative abundance and frequency (that is, potential specialist behavior) between either farming systems or SHTs, but we detected 1,001 OTUs strict specialists to farming systems or to SHTs (*multipatt*; the significance levels of *P* < 0.05, *P* < 0.01, and *P* < 0.001 were considered), representing 28.5% of the total OTU data set (3,507 OTUs). The taxonomic dendrograms (**Figure 4**; Supplementary Figure S1) illustrate the associations between OTUs and the farming systems and between OTUs and SHTs. Among 1,001 OTUs identified as habitat specialists, 836 OTUs (83.4%) were associated with conventional system (Supplementary Figure S1), 48 OTUs (4.8%) with organic system, 92 OTUs (9.2%) with a specific SHT and 25 OTUs (2.5%) with either farming system or SHT (**Figure 4**). The OTUs associated to farming systems or SHTs were broadly distributed across the phylogenetic tree with no deep or shallow taxonomic clades responding regularly to a specific management (Supplementary Figure S1; **Figure 4**). However, abundant members belonging to phyla *Proteobacteria* and *Acidobacteria* showed an accumulation of these habitat specialist OTUs. Notably, the Anaerobic soil disinfestation treatment constituted a contrast to the heterogeneous distributions of the taxonomic clades across soil treatments. On this treatment, habitat specific OTUs belonging to *Bacillales* and *Clostridialles* (*Firmicutes*) dominated the community (**Figure 4**).

## Discussion

The SHE represents a unique experiment to assess the response of microbial communities to farming systems (conventional and organic) and Soil Health Treatments (SHTs). This study was limited to temporal sampling (single time point), spatial extent (local scale), and therefore should not be generalized for the farming systems performed in all ecosystems. Although the consistent results in this study provide novel ecological insights into microbial ecology in agro-ecosystems, concrete conclusions are still difficult and need to be confirmed by long-term experiments over distinct environmental conditions, management practices and larger geographic scales. Besides this, the complexity of microbial communities and the technical constraints so far, limited our understanding of the relationship between soil microbiota and agricultural managements. However, using the approach based on high-throughput sequencing of amplified taxonomic markers, we have described the microbial community structure and found that the soil microbiome is more heterogeneous in organic than conventional farming system, and additionally identified potential microbial pathogen suppressors and individual microbial taxon associated with specific management practices.

It is difficult to draw robust and generalized conclusions on the effect of systems management on microbial diversity, but an increase in microbial diversity has been repeatedly observed in organic in comparison with conventional system (Mader et al., 2002; Hartmann et al., 2015). The increase of microbial diversity in organic systems is strongly associated with the management applied, including the organic amendments and practices related with reduction or absence of chemical inputs and biological plant protection (Sun et al., 2004; Chaudhry et al., 2012). The enhancement of microbial diversity also benefits the functional activities and a more heterogeneous distribution of species within the microbial assembly, which implies in a stable and functional redundant community, leading to an ecosystem functionality built on healthier interactions between the different trophic ecosystem levels (Brussaard et al., 2007; Postma et al., 2008; Crowder et al., 2010; Wagg et al., 2014). The decrease of microbial diversity in the conventional system may be explained by the direct or indirect long-term stresses caused by the use of pesticides, fungicides and herbicides used for plant protection. These agrochemicals reduce the total microbial diversity because of the potential to inhibit or eliminate certain groups of microbes and select members adapted or able to growth under conventional farming practices (El Fantroussi et al., 1999; Liu et al., 2007; Stagnari et al., 2014).

Our study revealed consistent farming system effects on microbial community variability, suggesting, for the first time, more heterogeneous community in organic than in conventional system. We suggest that the availability of rich substrate in soil through the introduction of cattle farm yard manure, the biological practices without the interference of synthetic compounds and the presence of weed species provide heterogeneous habitat niches, which can be occupied by a highly variable microbial community resulting in an increase of the beta-diversity. The lower heterogeneity (that is, the lower beta diversity) in microbial community in conventional system is an indication of biotic homogenization, the process of increasing similarity in the composition of communities across an array of taxonomic or functional groups (Olden et al., 2004). Biotic homogenization is a common pattern of the above-ground community in conventional systems (Gabriel et al., 2006), and recently was reported for microbial communities as a response to long-term cultivation (Montecchia et al., 2015). When poor agricultural practices are applied, such as uniformly crop monocultures, fertilization and intensive use of agrochemicals, the chain-reaction of (bio)diversity loss reduce the ecological niches leading to a homogenization of the microbial community and their functional gene pool, altering the ecosystem functioning and reducing the ecosystem resilience (Olden et al., 2004; Constancias et al., 2014; Figuerola et al., 2015). We acknowledge that the plant species planted in conventional and organic systems between 2006 and 2013 were not the same. This might have some impact on rhizosphere microbial community due to the different exudates released by different plant species. However, in this study we have focused on bulk soils and not on rhizosphere microbiome.

Besides the effects of farming systems on microbial community, we hypothesized that there is a legacy effects of the SHTs on diversity. It is expected that the differences between SHTs (e.g., organic matter composition, C/N, physical disturbances) may alter the physical, chemical and biological properties of the soil with consequent shifts in microbial diversity (Jacquiod et al., 2013). However, this study does not support evidence for the occurrence of long-term effects of SHTs on microbial diversity and richness. The first possible explanation is that different SHTs affects microbial diversity only in short-term and this effect may not be observed 3 years after the last application of the different treatments in this study. Some studies suggest a strong and fast resilience of the microbial diversity after a pronounced disturbance on soil community caused by management practices (Ding et al., 2014; Suleiman et al., 2016). Second, the continuous long-term farming system can counteract the effects of the soil health treatments, which were applied only twice. It has been suggested that long-term management practices are more likely to greatly influence the microbial community than temporal disturbances in soil (Buckley and Schmidt, 2003). Finally, we believe that the legacy effect of the SHTs occurs in specific microbial groups and cannot be resolved by determining the diversity and heterogeneity of entire microbial community, because shifts in some groups might be compensated by shifts in others.

It has been proposed that due to larger availability of organic carbon and nitrogen, organic system should favor copiotrophic bacteria, while oligotrophic should predominate in conventional systems, where the organic carbon quality is low (Ding et al., 2014; Hartmann et al., 2015). In this study, we observed that the differences in the structure of microbial communities between conventional and organic farming systems were mainly related to a large fraction of habitat specialist OTUs broadly dispersed across the phylogenetic groups belonging to almost all phyla found in soil. Only few taxonomic groups revealed to respond more uniformly to farming system. For example, most of habitat specialists assigned to *Proteobacteria* and *Euryarchaeota* were associated with conventional system and an increase of members belonging to *Acidobacteria* and *Planctomycetes* was detected in organic system. These findings are not necessarily surprising, but are an opposite trend toward the copiotrophic-oligotrophic categories expected. However, the rather dispersed OTU association between conventional and organic systems within these taxonomic groups are in agreement with the contrasting behavior of individual members within phyla reported previously (Rousk et al., 2010). Not all members in a taxonomic clade demonstrate the same ecological characteristics, implying that the general lifestyle classification might not be applied for all members in a phylum (Navarrete et al., 2013), and responses to management will occur at lower taxonomic levels rather than at major groups. *Proteobacteria* have been suggested to be a primarily copiotrophic phylum in soil (Li et al., 2012), while the lifestyle of microbial groups belonging to *Euryarchaeota*, which are predominately methanogens, are largely unknown (Angel et al., 2012). However, the increased abundance of taxa belonging to these two Phyla in conventional farming system may be promoted by the input of fertilizers, which create copiotrophic environment in nutrient-rich microhabitats and stimulate plant growth, enhancing carbon availability and favoring the growth rate of members of these phyla. Members of *Acidobacteria* and *Planctomyces* have been suggested to be adapted to nutrient-poor soils, and the input of organic amendments is expected to inhibit their activity (Buckley et al., 2006; Chaudhry et al., 2012). However, *Acidobacteria* and *Planctomyces* might be involved in the turnover of soil organic carbon and nutrient availability, pointing out that the manure addition in soil might promote the proliferation of these groups.

Microbial communities proved to be sensitive to SHTs. This is an important finding because microbial taxa strongly associated with management practices may help to elucidate the process behind soil suppressiveness. In previous study in the same SHE (Korthals et al., 2014), the SHTs were evaluated within conventional system on the potential effects on plant-parasitic nematode *P. penetrans* and soil-borne fungi *V. dahliae*. The combination, chitin, anaerobic soil disinfestation and marigold treatments were more effective in controlling *P. penetrans* and *V. dahliae* when compared with chemical control. In contrast, grass-clover, biofumigation, cultivit and compost were not effective alternatives. However, in that study, the bacterial community was not assessed. In this study, we revealed several taxa associated with SHTs distributed among major taxonomic groups, for which we have little or no information about their ecological roles. Therefore, we can only speculate the ecological importance of the detected taxa based on what has been described in previous studies and compare with findings on pathogen control (Korthals et al., 2014). A complete description of the results is beyond the scope of this study and we only focus on some consistent findings and their potential as soil microbe indicators for sustainable practices.

In anaerobic soil disinfestation treatment most of habitat specific OTUs were represented by taxa belonging to *Bacillales* and *Clostridialles* (*Firmicutes*), whose dominance is linked to their spore-forming capability, a competitive advantage under anaerobic conditions. Members belonging to family *Bacillales* have been described to be responsible for suppression of soil-borne disease-causing fungi (*Verticillium, Rhizoctonia* and *Fusarium*) and plant-parasitic nematodes (*Meloidogyne* and *Pratylenchus*) through production of antimicrobial compound and pore-forming toxins (crystal proteins) (Wei et al., 2003). Thus, this treatment selected *Firmicutes* taxa that might be involved in suppression of fungi and nematodes. In addition, habitat specific OTUs belonging to phylum *Nitrospira*, nitrite-oxidizing bacteria, were also associated with this treatment. This may be an indication of previous accumulation of ammonia (NH_3_) and production of nitrite (NO_2_), both nitrogenous compounds released due to decomposing of organic material known to play an important role in the suppression of fungi and nematodes (Tenuta and Lazarovits, 2002; Oka, 2010).

The genus *Lysobacter*, chitinolytic bacteria, was found to be associated with chitin treatment and have been described to have an important role in soil suppressiveness, with a potential antagonistic property against *Rhizoctonia* and nematodes plant pathogens (Tian et al., 2007; Postma et al., 2008). The genus *Virgibacillus*, another chitinolytic bacteria (Cretoiu et al., 2014), was also found to be associated with chitin treatment, but its role in soil suppressiveness is not described yet. Chitin is a major component of nematode egg shells and cell wall of most plant-pathogenic fungi, and it is assumed that chitin amendments increase the number of chitinolytic microorganisms and chitinase activity, which in turn suppress nematodes and fungi. Members of *Flavobacteriales* and *Chitinophagaceae* associated with marigold may also suppress soil nematodes by their chitinase activity (Glavina et al., 2010; Kharade and McBride, 2014), suggesting that besides its ability to produce nematicidal compounds, marigold can also recruit nematode-antagonistic microorganisms (Hooks et al., 2010).

The potential plant pathogens antagonists *Pasteuria, Pseudomonas* and *Burkholderiales* were associated with cultivit and grass-clover treatments. Bacterial taxa belonging to these groups have been described to be potential against plant-parasitic nematodes and fungi (Tian et al., 2007). However, our results suggest that multiple mechanisms may accounted for an effective soil suppressiveness and the simple presence of taxa with antagonistic behavior against plant pathogens is not a sufficient proof for successful suppression of a pathogen in soil (Weller et al., 2002). Thereafter, the alternative methods to control plant pathogens require more detailed studies in combination with molecular and traditional approaches used in plant pathology and microbiology.

Altogether our results indicate that conventional and organic farming systems had a major influence on soil diversity and composition of microbial communities while the effects of the SHTs were of smaller magnitude. Organic farming system promoted beneficial effects on biotic aspects regarding to microbial diversities, richness and community heterogeneity. However, the response of microbial community to farming systems is diverse and complex, and simple conclusions like “organic systems increased the soil biodiversity” may not be directly synonymous with concomitant increase in soil health and plant productivity. Furthermore, impact of the diversity losses in conventional system is not yet known; it is not clear how microbial diversity is related to ecosystem function and whether the changes in diversity we observed are reversible and the long-term consequences remain to be unexplored. Moreover, we detected that there is a legacy of the SHT which selects for treatment-specific microbial members that are consistent with the existing knowledge, but the limited phylogenetic and functional information precludes more definite conclusions about the beneficial impact of individual taxonomic groups with soil suppressiveness. However, the observed shifts in microbial diversity, community structure and individual taxon bring novel insights into the potential of managing the microbial community for sustainable agricultural productivity.

## Author Contributions

Design the experiment: GK and EK. Obtain and process the data: ML, TJ, and EK data. Analyze the data: ML and MH. Wrote the paper: ML and EK with contribution of all co-authors.

## Conflict of Interest Statement

The authors declare that the research was conducted in the absence of any commercial or financial relationships that could be construed as a potential conflict of interest.

## Footnotes

1 https://www.ebi.ac.uk/ena/
