# Supplementary material for: Soil Microbiome Is More Heterogeneous in Organic Than in Conventional Farming System
Source: Front Microbiol. 2017 Jan 4;7:2064. doi: 10.3389/fmicb.2016.02064 (PMC5209367; doi:10.3389/fmicb.2016.02064)
Supplement: Supplementary file 1 [file Data_Sheet_1.DOCX]

Soil microbiome is more heterogeneous in organic than conventional farming system

Manoeli Lupatini^1^, Gerard W. Korthals^2^, Mattias de Hollander^1^, Thierry K.S. Janssens^3^, Eiko E. Kuramae^1*^

^1^Department of Microbial Ecology, Netherlands Institute of Ecology (NIOO-KNAW), Wageningen, The Netherlands

^2^Department of Terrestrial Ecology, Netherlands Institute of Ecology (NIOO-KNAW), Wageningen, The Netherlands

^3^MicroLife Solutions, Amsterdam, The Netherlands

Running Head: Microbiome response to farming systems and soil health

*Address correspondence to Eiko E. Kuramae, [e.kuramae@nioo.knaw.nl](mailto:e.kuramae@nioo.knaw.nl).

Keywords: Soil Health treatment, Soil-borne pathogen, Sustainability, Agro-ecosystem, 16S rRNA, Bioindicator, Microbial Ecology, Microbial diversity

**Table S1.** Sample ID, Soil Health Treatments (SHTs) in conventional and organic systems, number of 16S rRNA partial gene sequences and Good's coverage used in this study.

| **Sample ID** | **SHT** | **System** | **Number of sequences*** | **Good's coverage**** |
| --- | --- | --- | --- | --- |
| Sample 74 | Anaerobic-soil-disinfestation | Conventional | 11673 | 0.87 |
| Sample 104 | Anaerobic-soil-disinfestation | Conventional | 18377 | 0.92 |
| Sample 15 | Anaerobic-soil-disinfestation | Conventional | 19021 | 0.90 |
| Sample 109 | Chemical-control | Conventional | 7532 | 0.86 |
| Sample 73 | Chemical-control | Conventional | 11911 | 0.88 |
| Sample 13 | Chemical-control | Conventional | 22875 | 0.93 |
| Sample 80 | Chitin | Conventional | 9944 | 0.89 |
| Sample 101 | Chitin | Conventional | 12378 | 0.88 |
| Sample 11 | Chitin | Conventional | 22918 | 0.91 |
| Sample 108 | Combination | Conventional | 9182 | 0.86 |
| Sample 79 | Combination | Conventional | 15386 | 0.92 |
| Sample 16 | Combination | Conventional | 16453 | 0.88 |
| Sample 103 | Compost | Conventional | 12120 | 0.90 |
| Sample 17 | Compost | Conventional | 13211 | 0.89 |
| Sample 75 | Compost | Conventional | 14592 | 0.90 |
| Sample 71 | Control | Conventional | 11228 | 0.88 |
| Sample 19 | Control | Conventional | 15708 | 0.91 |
| Sample 110 | Control | Conventional | 16538 | 0.91 |
| Sample 102 | Grass-clover | Conventional | 10643 | 0.88 |
| Sample 76 | Grass-clover | Conventional | 11217 | 0.88 |
| Sample 12 | Grass-clover | Conventional | 24389 | 0.92 |
| Sample 72 | Marigold | Conventional | 13467 | 0.91 |
| Sample 106 | Marigold | Conventional | 18208 | 0.89 |
| Sample 18 | Marigold | Conventional | 23461 | 0.93 |
| Sample 20 | PhysicalControl | Conventional | 10820 | 0.88 |
| Sample 77 | PhysicalControl | Conventional | 16404 | 0.90 |
| Sample 107 | PhysicalControl | Conventional | 17731 | 0.89 |
| Sample 14 | Biofumigation | Conventional | 0 | - |
| Sample 78 | Biofumigation | Conventional | 0 | - |
| Sample 105 | Biofumigation | Conventional | 287 | - |
| Sample 65 | Anaerobic-soil-disinfestation | Organic | 1884 | 0.68 |
| Sample 113 | Anaerobic-soil-disinfestation | Organic | 7607 | 0.85 |
| Sample 7 | Anaerobic-soil-disinfestation | Organic | 9184 | 0.85 |
| Sample 68 | Caliente | Organic | 5133 | 0.79 |
| Sample 111 | Caliente | Organic | 10845 | 0.88 |
| Sample 9 | Caliente | Organic | 14916 | 0.89 |
| Sample 64 | Chitin | Organic | 4322 | 0.80 |
| Sample 119 | Chitin | Organic | 4880 | 0.82 |
| Sample 2 | Chitin | Organic | 9098 | 0.87 |
| Sample 67 | Combination | Organic | 6262 | 0.83 |
| Sample 120 | Combination | Organic | 8886 | 0.83 |
| Sample 8 | Combination | Organic | 9276 | 0.87 |
| Sample 10 | Compost | Organic | 12273 | 0.87 |
| Sample 66 | Compost | Organic | 8651 | 0.84 |
| Sample 116 | Compost | Organic | 11144 | 0.87 |
| Sample 114 | Control | Organic | 2633 | 0.77 |
| Sample 62 | Control | Organic | 7306 | 0.83 |
| Sample 1 | Control | Organic | 13739 | 0.88 |
| Sample 118 | Grass-clover | Organic | 9432 | 0.85 |
| Sample 61 | Grass-clover | Organic | 5159 | 0.83 |
| Sample 5 | Grass-clover | Organic | 8076 | 0.86 |
| Sample 63 | Marigold | Organic | 4269 | 0.80 |
| Sample 6 | Marigold | Organic | 8217 | 0.85 |
| Sample 112 | Marigold | Organic | 8787 | 0.87 |
| Sample 69 | PhysicalControl | Organic | 6240 | 0.80 |
| Sample 4 | PhysicalControl | Organic | 9010 | 0.87 |
| Sample 115 | PhysicalControl | Organic | 10682 | 0.86 |
| Sample 3 | Biofumigation | Organic | 20 | - |
| Sample 70 | Biofumigation | Organic | 212 | - |
| Sample 117 | Biofumigation | Organic | 139 | - |

* The number after reads processing and removal of Cyanobacteria_Chloroplast, Mitochondria_genus_incertae_sedis, unknown and unclassified.

** Good's estimator of coverage was calculated using the formula: (1−(singletons/individuals)) × 100 only for Bacteria and Archaea Domain.

**Table S2.** Relative abundance of soil bacterial and archaeal phyla in Soil Health Treatments (SHTs) and conventional and organic farming systems

|  | **Farming System** | | | | | | | | | | | | | | | | | | **Statistics** | | | |
| --- | --- | --- | --- | --- | --- | --- | --- | --- | --- | --- | --- | --- | --- | --- | --- | --- | --- | --- | --- | --- | --- | --- |
|  | **Conventional** | | | | | | | | | **Organic** | | | | | | | | |  |  |  |  |
| **Phylum/SHT** | **Anaerobic-soil-disinfestation** | **Chemical-control** | **Chitin** | **Combination** | **Compost** | **Control** | **Grass-clover** | **Marigold** | **Physical Control** | **Anaerobic-soil-disinfestation** | **Caliente** | **Chitin** | **Combination** | **Compost** | **Control** | **Grass-clover** | **Marigold** | **Physical Control** | **System** | **Treatment** | **System * Treatment** | **Block** |
| ***Proteobacteria*** | 0.51^a^ (0.03)  **A**^c^ | 0.42  (0.13)  **A** | 0.56  (0.30)  **A** | 0.51  (0.01)  **A** | 0.54  (0.03)  **A** | 0.50  (0.10)  **A** | 0.52  (0.04)  **A** | 0.55  (0.08)  **A** | 0.46  (0.02)  **A** | 0.50  (0.03)  **B** | 0.47  (0.08)  **B** | 0.43  (0.11)  **B** | 0.42  (0.12)  **B** | 0.51  (0.03)  **B** | 0.46  (0.07)  **B** | 0.48  (0.06)  **B** | 0.52  (0.07)  **B** | 0.48  (0.08)  **B** | *^b^ | ns | ns | * |
| ***Firmicutes*** | 0.11  (0.04)  **a** | 0.05  (0.01)  **b** | 0.07  (0.04)  **ab** | 0.09  (0.02)  **ab** | 0.06  (0.03)  **ab** | 0.05  (0.01)  **b** | 0.05  (0.01)  **ab** | 0.05  (0.01)  **b** | 0.06  (0.01)  **ab** | 0.08  (0.01) | 0.04  (0.00) | 0.07  (0.01) | 0.06  (0.01) | 0.08  (0.02) | 0.04  (0.01) | 0.09  (0.03) | 0.05  (0.01) | 0.06  (0.00) | ns | *** | ● | ns |
| ***Acidobacteria*** | 0.12  (0.04)  **B** | 0.21  (0.1)  **B** | 0.10  (0.01)  **B** | 0.12  (0.02)  **B** | 0.12  (0.01)  **B** | 0.12  (0.03)  **B** | 0.15  (0.03)  **B** | 0.12  (0.06)  **B** | 0.19  (0.03)  **B** | 0.16  (0.03)  **A** | 0.19  (0.07)  **A** | 0.19  (0.07)  **A** | 0.21  (0.07)  **A** | 0.16  (0.05)  **A** | 0.21  (0.02)  **A** | 0.13  (0.04)  **A** | 0.15  (0.03)  **A** | 0.19  (0.06)  **A** | *** | ● | ns | ** |
| ***Bacteroidetes^d^*** | 0.10  (0.03) | 0.08  (0.02) | 0.10  (0.03) | 0.11  (0.03) | 0.11  (0.02) | 0.14  (0.07) | 0.08  (0.02) | 0.11  (0.02) | 0.07  (0.00) | 0.08  (0.00) | 0.09  (0.01) | 0.08  (0.01) | 0.08  (0.01) | 0.07  (0.00) | 0.09  (0.02) | 0.10  (0.00) | 0.10  (0.03) | 0.08  (0.02) | * | ns | ns | * |
| ***Actinobacteria*** | 0.04  (0.01) | 0.04  (0.01) | 0.04  (0.01) | 0.05  (0.00) | 0.04  (0.01) | 0.05  (0.00) | 0.04  (0.01) | 0.05  (0.00) | 0.04  (0.01) | 0.05  (0.01) | 0.04  (0.02) | 0.05  (0.03) | 0.04  (0.02) | 0.05  (0.02) | 0.04  (0.01) | 0.05  (0.01) | 0.04  (0.01) | 0.05  (0.03) | ns | ns | ns | ns |
| ***Verrucomicrobia*** | 0.02  (0.01) | 0.03  (0.01) | 0.02  (0.01) | 0.02  (0.01) | 0.02  (0.00) | 0.02  (0.01) | 0.02  (0.01) | 0.02  (0.02) | 0.03  (0.00) | 0.02  (0.00) | 0.03  (0.01) | 0.02  (0.01) | 0.03  (0.01) | 0.02  (0.01) | 0.03  (0.01) | 0.02  (0.01) | 0.02  (0.01) | 0.03  (0.02) | ns | ns | ns | ns |
| **unclassified_A** | 4.47  (2.10)  x 10^-3^  **A** | 3.37  (2.03)  x 10^-3^  **A** | 2.95  x 10^-3^  (9.36  x 10^-4^) **A** | 2.38  (2.36)  x 10^-3^  **A** | 4.04  (2.34)  x 10^-3^  **A** | 2.11  (1.92)  x 10^-3^  **A** | 6.51  (5.89)  x 10^-3^  **A** | 3.52  (4.45)  x 10^-3^  **A** | 6.88  (7.60)  x 10^-3^  **A** | 1.48  (1.42)  x 10^-3^  **B** | 1.50  x 10^-3^  (9.23  x 10^-4^)  **B** | 1.17  x 10^-3^  (8.21  x 10^-4^)  **B** | 1.57  (1.12)  x 10^-3^  **B** | 1.47  (1.98)  x 10^-3^  **B** | 2.75  (2.99)  x 10^-3^  **B** | 1.34  (2.14)  x 10^-3^  **B** | 4.60  (2.87)  x 10^-3^  **B** | 2.97  (2.30)  x 10^-3^  **B** | * | ns | ns | * |
| ***Crenarchaeota*** | 2.78  (1.15)  x 10^-3^ | 2.38  (1.98)  x 10^-3^ | 1.96  x 10^-3^  (9.07  x 10^-4^) | 2.76  (1.98)  x 10^-3^ | 4.30  (1.99)  x 10^-3^ | 1.71  x 10^-2^  (0.02) | 4.17  (2.25)  x 10^-3^ | 3.36  (4.08)  x 10^-3^ | 3.35  (2.87)  x 10^-3^ | 6.21  x 10^-3^  (5.78  x 10^-4)^ | 1.62  x 10^-3^  (8.96  x 10^-4)^ | 1.44  x 10^-3^  (8.32  x 10^-4^) | 1.89  x 10^-3^  (2.58  x 10^-4)^ | 2.02  (1.72)  x 10^-3^ | 6.11  (5.79)  x 10^-3^ | 7.93  x 10^-3^  (0.01) | 2.13  (1.22)  x 10^-3^ | 2.29  (1.93)  x 10^-3^ | ns | ns | ns | ns |
| **unclassified_B** | 0.08  (0.01)  **B** | 0.13  (0.07)  **B** | 0.07  (2.94  x 10^-3^)  **B** | 0.07  (0.01)  **B** | 0.08  (4.30  x 10^-3^)  **B** | 0.08  (0.02)  **B** | 0.10  (0.01)  **B** | 0.07  (0.01)  **B** | 0.12  (0.03)  **B** | 0.09  (0.01)  **A** | 0.10  (0.01)  **A** | 0.11  (0.02)  **A** | 0.13  (0.03)  **A** | 0.09  (3.80  x 10^-3^)  **A** | 0.09  (0.02)  **A** | 0.09  (0.02)  **A** | 0.08  (0.01)  **A** | 0.09  (0.01)  **A** | ** | ns | * | ns |
| ***Gemmatimonadetes*** | 7.27  (1.04)  x 10^-3^ | 7.41  (1.24)  x 10^-3^ | 9.28  x 10^-3^  (7.60  x 10^-4^) | 5.03  (2.56)  x 10^-3^ | 6.38  (2.10)  x 10^-3^ | 5.01  (3.16)  x 10^-3^ | 7.02  x 10^-3^  (8.39  x 10^-4^) | 6.53  x 10^-3^  (2.07  x 10^-4^) | 6.33  (1.50)  x 10^-3^ | 8.39  (1.25)  x 10^-3^ | 7.32  (3.54)  x 10^-3^ | 9.13  (3.92)  x 10^-3^ | 5.66  (4.00)  x 10^-3^ | 7.55  (2.77)  x 10^-3^ | 6.38  (4.43)  x 10^-3^ | 8.73  x 10^-3^  (9.70  x 10^-4^) | 7.67  (3.44)  x 10^-3^ | 7.52  (1.91)  x 10^-3^ | ns | ns | ns | * |
| ***Planctomycetes*** | 6.47  (1.70)  x 10^-3^  **B** | 0.01  (6.81  x 10^-3^)  **B** | 3.45  x 10^-3^  (6.21  x 10^-4^)  **B** | 5.31  (3.34)  x 10^-3^  **B** | 7.30  (3.02)  x 10^-3^  **B** | 7.92E  (6.14)  x 10^-3^  **B** | 9.04  (4.03)  x 10^-3^  **B** | 5.64  (5.43)  x 10^-3^  **B** | 0.01  (2.15  x 10^-3^)  **B** | 0.01  (4.47  x 10^-3^)  **A** | 0.01  (4.36  x 10^-3^)  **A** | 0.01  (8.78  x 10^-3^)  **A** | 0.01  (7.05  x 10^-3^)  **A** | 6.95  x 10^-3^  (1.92  x 10^-3^)  **A** | 0.01  (5.74  x 10^-3^)  **A** | 8.00  x 10^-3^  (3.59  x 10^-3^)  **A** | 0.01  (7.94  x 10^-3^)  **A** | 8.66  x 10^-3^  (2.84  x 10^-3^)  **A** | * | ns | ns | ● |
| ***Nitrospira*** | 1.28  x 10^-3^  (6.42  x 10^-4^)  **a** | 1.23  x 10^-3^  (4.41  x 10^-4)^  **b** | 2.87  (1.30)  x 10^-3^  **ab** | 1.12  x 10^-3^  (1.95  x 10^-4)^  **ab** | 1.54  (1.00)  x 10^-3^  **ab** | 1.27  x 10^-3^  (4.96  x 10^-4)^  **b** | 2.13  x 10^-3^  (1.03  x 10^-4)^  **b** | 9.53  x 10^-4^  (2.19  x 10^-4)^  **b** | 1.15  x 10^-3^  (3.86  x 10^-4)^  **ab** | 1.24  (1.00)  x 10^-3^ | 1.68  x 10^-3^  (2.58  x 10^-4)^ | 2.70  x 10^-3^  (6.09  x 10^-4)^ | 1.91  x 10^-3^  (7.63  x 10^-4)^ | 1.47  x 10^-3^  (2.93  x 10^-4)^ | 1.14  x 10^-3^  (1.61  x 10^-4)^ | 8.25x 10^-4^  (5.40  x 10^-4)^ | 5.55  x 10^-4^  (2.62  x 10^-4)^ | 1.98  x 10^-3^  (4.80  x 10^-4)^ | ns | *** | * | * |
| ***Chloroflexi*** | 1.10  x 10^-3^  (4.29  x 10^-4^) | 2.67  (3.20)  x 10^-3^ | 6.17  x 10^-4^  (6.55  x 10^-4^) | 2.12  (1.60)  x 10^-3^ | 1.63  (1.44)  x 10^-3^ | 1.41  x 10^-3^  (6.66  x 10^-4^) | 1.49  x 10^-3^  (7.20  x 10^-4^) | 4.02x 10^-4^  (4.69  x 10^-5^) | 1.84  x 10^-3^  (1.31  x 10^-4^) | 6.40  (5.57)  x 10^-4^ | 9.02  (6.97)  x 10^-4^ | 6.13  (7.13)  x 10^-3^ | 4.10  (2.68)  x 10^-3^ | 1.93  (1.75)  x 10^-3^ | 1.92  (1.73)  x 10^-3^ | 1.95  (1.96)  x 10^-3^ | 1.22  x 10^-3^  (8.67  x 10^-4^) | 7.48  x 10^-4^  (1.66  x 10^-4^) | ns | ns | ns | ns |
| ***Spirochaetes*** | 6.40  (4.91)  x 10^-4^ | 6.57  (3.13)  x 10^-4^ | 2.26  (1.18)  x 10^-4^ | 3.24  (1.17)  x 10^-4^ | 1.07  (1.13)  x 10^-3^ | 3.69  (1.56)  x 10^-4^ | 6.51  (5.76)  x 10^-4^ | 6.57  (4.57)  x 10^-4^ | 3.39  (1.56)  x 10^-4^ | 2.85  (3.09)  x 10^-4^ | 6.07  (2.86)  x 10^-4^ | 7.42  (2.17)  x 10^-4^ | 7.99  (3.92)  x 10^-4^ | 3.35x 10^-4^  (6.82  x 10^-5^) | 8.34  (6.13)  x 10^-4^ | 8.29  (5.94)  x 10^-4^ | 1.48  x 10^-3^  (5.19  x 10^-4^) | 3.71  (1.38)  x 10^-4^ | ns | ns | ns | ns |
| ***Armatimonadetes*** | 2.83  x 10^-3^  (9.51  x 10^-4^) | 3.80  x 10^-3^  (7.82  x 10^-4^) | 3.16  x 10^-3^  (8.46  x 10^-4^) | 2.37  x 10^-3^  (3.59  x 10^-4^) | 3.20  (1.00)  x 10^-3^ | 3.69  x 10^-3^  (2.29  x 10^-4^) | 2.98  x 10^-3^  (5.28  x 10^-4^) | 3.50  x 10^-3^  (5.38  x 10^-4^) | 4.37  x 10^-3^  (5.15  x 10^-4^) | 2.68  x 10^-3^  (8.68  x 10^-4^) | 3.52  (1.03)  x 10^-3^ | 3.87  x 10^-3^  (3.32  x 10^-4^) | 3.61  (1.24)  x 10^-3^ | 3.36  (1.03)  x 10^-3^ | 2.78  x 10^-3^  (1.45  x 10^-4^) | 3.12  x 10^-3^  (5.49  x 10^-4^) | 3.36  x 10^-3^  (6.94  x 10^-4^) | 3.85  (1.35)  x 10^-3^ | ns | ns | ns | ns |
| ***WS3*** | 3.31  (2.83)  x 10^-4^  **abc** | 1.12  x 10^-3^  (9.17  x 10^-4^)  **ab** | 8.07  (3.06)  x 10^-5^  **bc** | 1.68  (1.46)  x 10^-4^  **abc** | 1.39  (1.08)  x 10^-4^  **abc** | 1.33  (1.15)  x 10^-4^  **bc** | 5.38  (4.18)  x 10^-4^  **abc** | 1.15  (1.62)  x 10^-4^  **c** | 1.09  x 10^-3^  (3.44)  x 10^-4^)  **a** | 4.35  (2.74)  x 10^-4^ | 5.25  (4.31)  x 10^-4^ | 0.00  (0.00) | 5.90  (4.92)  x 10^-4^ | 5.09  (2.53)  x 10^-4^ | 2.88  (2.78)  x 10^-4^ | 1.20  (2.07)  x 10^-4^ | 2.94  (1.83)  x 10^-4^ | 4.53  (3.31)  x 10^-4^ | ns | *** | * | ● |
| ***Euryarchaeota*** | 3.55  (2.44)  x 10^-4^  **A** | 3.76  (1.98)  x 10^-4^  **A** | 4.50  (4.38)  x 10^-5^  **A** | 3.39  (1.34)  x 10^-4^  **A** | 8.82  x 10^-5^  (1.06  x 10^-3^)  **A** | 1.06  x 10^-3^  (5.45  x 10^-4^)  **A** | 4.50  (5.79)  x 10^-4^  **A** | 2.34  (7.04)  x 10^-4^  **A** | 4.29  (1.15)  x 10^-4^  **A** | 2.05  (3.54)  x 10^-4^  **B** | 2.35  x 10^-4^  (4.67  x 10^-5^)  **B** | 2.48  (4.29)x 10^-4^  **B** | 1.96  (1.31)  x 10^-4^  **B** | 2.37  (1.31)  x 10^-4^  **B** | 2.07  (2.34)  x 10^-4^  **B** | 8.55  (9.13)  x 10^-4^  **B** | 2.97  (4.12)  x 10^-4^  **B** | 3.42  (5.93)  x 10^-5^  **B** | ** | ns | ns | ns |
| ***Deinococcus-Thermus^d^*** | 1.76  x 10^-4^  (5.70  x 10^-4^) | 3.27  (2.58)  x 10^-4^ | 5.77  (2.59)  x 10^-4^ | 5.68  (2.28)  x 10^-4^ | 2.25  (1.91)  x 10^-4^ | 5.77  (4.36)  x 10^-4^ | 4.01  (1.79)  x 10^-4^ | 2.11  (1.69)  x 10^-4^ | 1.55  (2.13)  x 10^-4^ | 7.99  x 10^-5^  (1.38  x 10^-4^) | 1.54  x 10^-4^  (6.08  x 10^-5^) | 1.08  x 10^-3^  (8.30  x 10^-4^) | 4.89  (2.24)  x 10^-4^ | 6.47  x 10^-5^  (1.12  x 10^-4^) | 4.18  (3.98)  x 10^-4^ | 7.83  (8.09)  x 10^-4^ | 2.13  (1.91)  x 10^-4^ | 1.14  (1.19)  x 10^-4^ | ns | ** | ns | ns |
| ***Chlamydiae*** | 2.77  (1.42)  x 10^-4^ | 1.91  (2.21)  x 10^-4^ | 4.83  x 10^-4^  (4.99  x 10^-5^) | 2.42  (1.44)  x 10^-4^ | 2.07  (1.45)  x 10^-4^ | 1.52  (1.61)  x 10^-4^ | 3.00  (3.06)  x 10^-4^ | 2.08  x 10^-4^  (4.42  x 10^-5^) | 2.71  (1.85)  x 10^-4^ | 4.38  (3.84)  x 10^-4^ | 2.11  x 10^-4^  (1.12  x 10^-5^) | 2.40  (2.37)  x 10^-4^ | 3.54  (1.06)  x 10^-4^ | 2.02  (2.76)  x 10^-4^ | 1.32  (2.29)  x 10^-4^ | 3.98  x 10^-5^  (6.90  x 10^-5^) | 2.16  x 10^-4^  (8.03  x 10^-5^) | 2.00  (1.84)  x 10^-4^ | ns | ns | ns | ns |
| ***Tenericutes^e^*** | 1.15  (1.99)  x 10^-4^ | 0.00  (0.00) | 0.00  (0.00) | 6.19  (5.95)  x 10^-5^ | 1.13  (9.79)  x 10^-4^ | 3.26  (5.65)  x 10^-5^ | 0.00  (0.00) | 1.06  (1.83)  x 10^-4^ | 0.00  (0.00) | 2.05  (3.54)  x 10^-4^ | 2.40  (4.16)  x 10^-5^ | 0.00  (0.00) | 0.00  (0.00) | 0.00  (0.00) | 0.00  (0.00) | 0.00  (0.00) | 0.00  (0.00) | 0.00  (0.00) | ns | ns | ns | ns |
| ***BRC1^e^*** | 0.00  (0.00) | 4.57  (4.54)  x 10^-5^ | 1.58  (2.73)  x 10^-5^ | 2.30  (3.98)  x 10^-5^ | 5.37  (4.71)  x 10^-5^ | 0.00  (0.00) | 1.27  (5.70)  x 10^-5^ | 4.00  (6.92)  x 10^-5^ | 1.11  (1.93)  x 10^-4^ | 0.00  (0.00) | 0.00  (0.00) | 0.00  (0.00) | 3.87  (6.70)  x 10^-5^ | 0.00  (0.00) | 2.07  (2.34)  x 10^-4^ | 0.00  (0.00) | 0.00  (0.00) | 3.96  (6.86)  x 10^-5^ | ns | ns | ns | ns |

^a^ The average based on three triplicate samples in each SHT within conventional and organic systems. The values between brackets are the standard deviation (n=3).

^b^ Significance levels for ANOVA test: ns = not significant (*P* > 0.1);‘^●^’ *P* < 0.1; ‘*’ P < 0.05; ‘**’P < 0.01 and ‘***’P < 0.001.

^c^ The uppercase letters indicate significant differences between conventional and organic system; lowercase letters indicate significant differences between SHT within conventional and organic systems. Values with the different letters were significantly different (P < 0.05) based on upon a Tukey's HSD test.

^d^ For *Bacteroidetes* and *Deinococcus-Thermus*, ANOVA show significance (*P* < 0.01), but pairwise comparison using Tukey's HSD test was not significant (*P* > 0.05).

^e^ It was not possible to reach the normal distribution of the residual for *Tenericutes* and BRC. Thus, the effects of the variables were tested by the Kruskal-Wallis test analysis using *kruskal.test* in “stats” package.


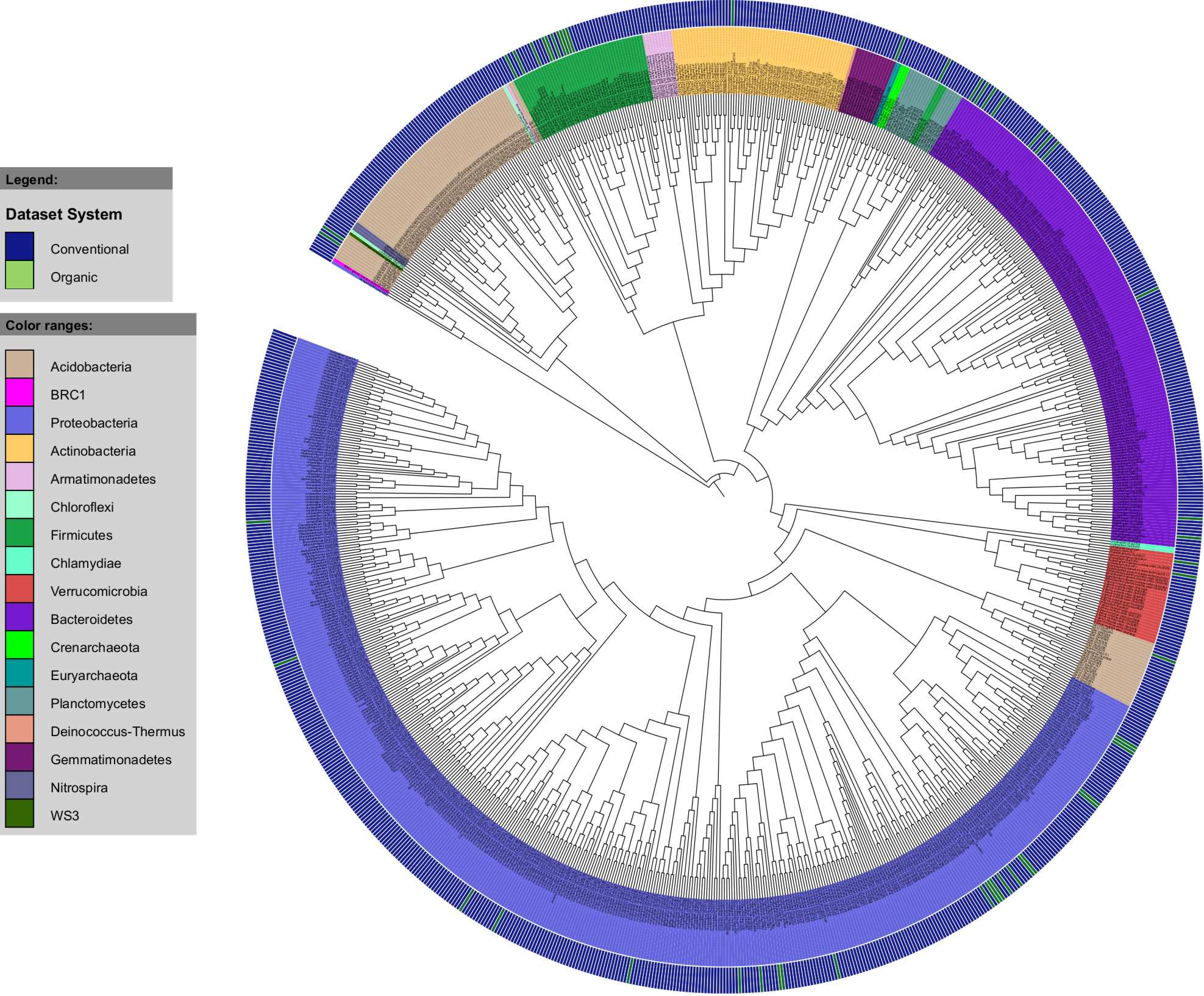


**Figure S1.** Dendrogram showing the taxonomy and the habitat specialists associated with conventional and organic farming systems. Only the strict specialists OTUs - cut-off 97% - (28.5% of the total OTU data) with statistical significance (*P* < 0.05, *P* < 0.01 and *P* < 0.001) were considered. The taxonomic affiliation at phylum level of each specialist OTU is identified by the colors range in the left panel and within the tree. The habits preference for a given OTU is indicated in the bars outside of the tree. The conventional framing system is represented by blue color and organic farming system by green color. More information about abundance of each OTU is provided as supplementary information.
